# Bis[μ-*N*′-acetyl-1-oxidonaphthalene-2-carbohydrazidato(3−)]tetra­pyridine­tricopper(II)

**DOI:** 10.1107/S160053680904389X

**Published:** 2009-10-28

**Authors:** Jin-Ping Gao, Wen Zhang, Xue-Feng Shi, Da-Cheng Li

**Affiliations:** a College of Chemistry and Chemical Engineering, Liaocheng University, Shandong 252059, People’s Republic of China

## Abstract

There are two half-mol­ecules in the asymmetric unit of the title compound, [Cu_3_(C_13_H_9_N_2_O_3_)_2_(C_5_H_5_N)_4_], and crystallographic inversion symmetry completes each trinuclear mol­ecule. In both mol­ecules, the central Cu atom (site symmetry 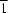) adopts a distorted *trans*-CuO_2_N_4_ octa­hedral geometry, arising from its coordination by two *N*,*O*-bidentate aroylhydrazine ligands and two pyridine mol­ecules. The peripheral Cu atoms adopt *trans*-CuN_2_O_2_ square-planar coordinations arising from an *N*,*O*,*O*-tri­dentate ligand (that also bonds to the central Cu atom) and a pyridine mol­ecule.

## Related literature

For related compounds, see: Patole *et al.* (2003 ▶); Pouralimardan *et al.* (2007 ▶). 
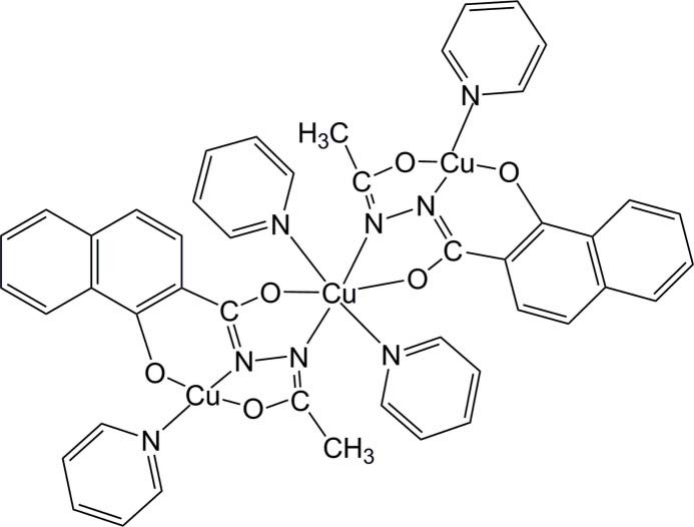


## Experimental

### 

#### Crystal data

[Cu_3_(C_13_H_9_N_2_O_3_)_2_(C_5_H_5_N)_4_]
*M* *_r_* = 989.47
Monoclinic, 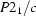

*a* = 24.080 (3) Å
*b* = 9.8572 (11) Å
*c* = 19.373 (2) Å
β = 111.371 (2)°
*V* = 4282.1 (8) Å^3^
*Z* = 4
Mo *K*α radiation
μ = 1.54 mm^−1^
*T* = 298 K
0.34 × 0.33 × 0.22 mm

#### Data collection

Bruker SMART CCD diffractometer
Absorption correction: multi-scan (*SADABS*; Bruker, 2003 ▶) *T*_min_ = 0.623, *T*_max_ = 0.729
21184 measured reflections
7546 independent reflections
3457 reflections with *I* > 2σ(*I*)
*R*_int_ = 0.071

#### Refinement

*R*[*F* ^2^ > 2σ(*F* ^2^)] = 0.053
*wR*(*F* ^2^) = 0.112
*S* = 1.00
7546 reflections
573 parameters
H-atom parameters constrained
Δρ_max_ = 0.87 e Å^−3^
Δρ_min_ = −0.45 e Å^−3^

### 

Data collection: *SMART* (Bruker, 2003 ▶); cell refinement: *SAINT* (Bruker, 2003 ▶); data reduction: *SAINT*; program(s) used to solve structure: *SHELXS97* (Sheldrick, 2008 ▶); program(s) used to refine structure: *SHELXL97* (Sheldrick, 2008 ▶); molecular graphics: *SHELXTL* (Sheldrick, 2008 ▶); software used to prepare material for publication: *SHELXTL*.

## Supplementary Material

Crystal structure: contains datablocks I, global. DOI: 10.1107/S160053680904389X/hb5100sup1.cif

Structure factors: contains datablocks I. DOI: 10.1107/S160053680904389X/hb5100Isup2.hkl

Additional supplementary materials:  crystallographic information; 3D view; checkCIF report

## Figures and Tables

**Table 1:** Selected bond lengths (Å)

Cu1—O1	2.022 (3)
Cu1—N2	2.061 (4)
Cu1—N3	2.172 (5)
Cu2—N1	1.832 (4)
Cu2—O2	1.838 (4)
Cu2—O3	1.875 (4)
Cu2—N4	1.951 (5)
Cu3—O4	2.025 (3)
Cu3—N6	2.068 (4)
Cu3—N7	2.176 (5)
Cu4—O5	1.827 (4)
Cu4—N5	1.828 (4)
Cu4—O6	1.861 (4)
Cu4—N8	1.944 (4)

**Fig. 1.**
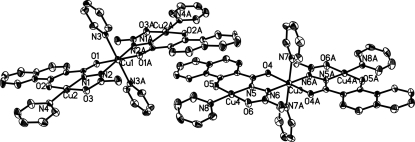
The molecular structure of (I) showing 30% probability displacement ellipsoids. H atoms have been omitted for clarity.

## supplementary crystallographic information

### Comment

A large number of aroylhydrazine complexes have been prepared and studied due to their diverse molecular architectures and quite interesting chemical properties. However, researches on the copper(II) complexes with *N*-acetyl-1 -hydroxy-2-naphthoylhydrazide have not reported. So we have synthesized a new complex(Fig.1), which has been characterized by X-ray diffraction and elemental analysis. The triple-deprotonated *N*-acetyl-1-hydroxy-2- naphthoylhydrazide bridged the metal ions using hydrazide N—N group and formed the trinuclear copper complex. As can be seen from Fig.1, there are two trinuclear molecules in the unit cell, molecule A and B. Three copper(II) ions are in a straight line with the Cu—Cu—Cu interatomic angle 180 (12)° for A and B. The atomic distance of Cu1—Cu2 and Cu3—Cu4 is 0.4609 nm and 0.491 nm. The coordination geometry of the three Cu atoms exhibit alternating square-planar and octahedral coordination mode. The copper atoms of the center (Cu1 and Cu3) exhibit distorted N_4_O_2_ octahedral surroundings. However, for the other terminal copper atoms (Cu2 and Cu4), the coordinationg geometry are square plane. In the crystal packing, the complex molecules are linked into one-dimensional chains by intermolecular C—H···O interactions (Fig. 2)

### Experimental

Acetic anhydride (0.52 g, 4 mmol) and 1-hydroxy-2-naphthoylhydrazide (0.808 g, 4 mmol)were added to 40 ml of chloroform at ice-water bath. The reaction mixture was slowly warmed to room temperature and stirred for 24 h. After staying for overnight at refrigerator,the resulting white precipitate was filtered and rinsed with chloroform and diethyl ether m.p.:162–167. An amount of 24.4 mg (0.1 mmol) of *N*-acetyl-1-hydroxy-2- naphthoylhydrazide was dissolved in 10 ml pyridine and 29.9 mg (0.15 mmol) of copper acetate was dissolved in 10 ml DMF. The two solutions were mixed and the combined solution was allowed to stand for 3 weeks, whereupon green blocks of (I) were obtained in 56% yield. Elemental analysis calculated for Cu_6_C_92_H_76_N_16_O_12_: C, 55.84; H, 3.87; N, 11.12. Found (%): C, 55.42; H, 4.06; N, 11.47

### Refinement

The H atoms were positioned with idealized geometry (C—H = 0.93–0.96 Å) and refined as riding with *U*_iso_(H) = 1.2 *U*_eq_(C) or 1.5U_eq_(methyl C).

### Crystal data

**Table:**

[Cu_3_(C_13_H_9_N_2_O_3_)_2_(C_5_H_5_N)_4_]	*F*(000) = 2020
*M**_r_* = 989.47	*D*_x_ = 1.535 Mg m^−^^3^
Monoclinic, *P*2_1_/*c*	Mo *K*α radiation, λ = 0.71073 Å
Hall symbol: -P 2ybc	Cell parameters from 1024 reflections
*a* = 24.080 (3) Å	θ = 2.4–19.6°
*b* = 9.8572 (11) Å	µ = 1.54 mm^−^^1^
*c* = 19.373 (2) Å	*T* = 298 K
β = 111.371 (2)°	Block, green
*V* = 4282.1 (8) Å^3^	0.34 × 0.33 × 0.22 mm
*Z* = 4	

### Data collection

**Table:**

Bruker SMART CCD diffractometer	7546 independent reflections
Radiation source: fine-focus sealed tube	3457 reflections with *I* > 2σ(*I*)
graphite	*R*_int_ = 0.071
ω scans	θ_max_ = 25.0°, θ_min_ = 1.8°
Absorption correction: multi-scan (*SADABS*; Bruker, 2003)	*h* = −28→28
*T*_min_ = 0.623, *T*_max_ = 0.729	*k* = −11→11
21184 measured reflections	*l* = −23→19

### Refinement

**Table:**

Refinement on *F*^2^	Primary atom site location: structure-invariant direct methods
Least-squares matrix: full	Secondary atom site location: difference Fourier map
*R*[*F*^2^ > 2σ(*F*^2^)] = 0.053	Hydrogen site location: inferred from neighbouring sites
*wR*(*F*^2^) = 0.112	H-atom parameters constrained
*S* = 1.00	*w* = 1/[σ^2^(*F*_o_^2^) + (0.0278*P*)^2^] where *P* = (*F*_o_^2^ + 2*F*_c_^2^)/3
7546 reflections	(Δ/σ)_max_ < 0.001
573 parameters	Δρ_max_ = 0.87 e Å^−^^3^
0 restraints	Δρ_min_ = −0.45 e Å^−^^3^

### Fractional atomic coordinates and isotropic or equivalent isotropic displacement parameters (Å^2^)

**Table:**

	*x*	*y*	*z*	*U*_iso_*/*U*_eq_	
Cu1	0.5000	1.0000	0.5000	0.0453 (3)	
Cu2	0.65591 (3)	0.82431 (8)	0.44280 (4)	0.0529 (2)	
Cu3	0.0000	0.5000	0.5000	0.0500 (3)	
Cu4	0.17821 (3)	0.50169 (8)	0.46069 (4)	0.0508 (2)	
N1	0.60961 (18)	0.9153 (4)	0.4845 (3)	0.0399 (12)	
N2	0.54793 (18)	0.8834 (5)	0.4530 (3)	0.0394 (12)	
N3	0.4999 (2)	1.1643 (5)	0.4252 (3)	0.0446 (13)	
N4	0.6990 (2)	0.7167 (5)	0.3940 (3)	0.0478 (13)	
N5	0.11599 (18)	0.5382 (4)	0.4906 (2)	0.0388 (12)	
N6	0.06561 (18)	0.4543 (5)	0.4584 (3)	0.0418 (13)	
N7	−0.0429 (2)	0.6330 (6)	0.4059 (3)	0.0501 (14)	
N8	0.2408 (2)	0.4511 (5)	0.4246 (3)	0.0451 (13)	
O1	0.58439 (15)	1.0504 (3)	0.5647 (2)	0.0426 (10)	
O2	0.72474 (15)	0.9138 (4)	0.4980 (2)	0.0481 (11)	
O3	0.58364 (16)	0.7416 (4)	0.3857 (2)	0.0513 (11)	
O4	0.06049 (15)	0.6467 (3)	0.5487 (2)	0.0465 (10)	
O5	0.22834 (15)	0.6264 (4)	0.5221 (2)	0.0474 (11)	
O6	0.12637 (16)	0.3725 (4)	0.4011 (2)	0.0538 (11)	
C1	0.6234 (2)	1.0023 (6)	0.5407 (3)	0.0372 (13)	
C2	0.7310 (2)	1.0014 (6)	0.5514 (3)	0.0466 (15)	
C3	0.6864 (2)	1.0462 (6)	0.5754 (3)	0.0405 (15)	
C4	0.6999 (3)	1.1336 (6)	0.6364 (3)	0.0514 (17)	
H4	0.6697	1.1590	0.6530	0.062*	
C5	0.7568 (3)	1.1829 (6)	0.6727 (4)	0.0651 (19)	
H5	0.7647	1.2396	0.7136	0.078*	
C6	0.8031 (3)	1.1473 (7)	0.6477 (4)	0.0574 (18)	
C7	0.7914 (3)	1.0603 (6)	0.5877 (4)	0.0483 (16)	
C8	0.8371 (3)	1.0303 (6)	0.5627 (4)	0.066 (2)	
H8	0.8304	0.9687	0.5241	0.079*	
C9	0.8920 (3)	1.0899 (8)	0.5939 (4)	0.078 (2)	
H9	0.9216	1.0717	0.5749	0.093*	
C10	0.9035 (3)	1.1760 (8)	0.6526 (5)	0.084 (2)	
H10	0.9411	1.2149	0.6737	0.101*	
C11	0.8610 (3)	1.2056 (7)	0.6805 (4)	0.073 (2)	
H11	0.8694	1.2637	0.7208	0.087*	
C12	0.5393 (2)	0.7935 (6)	0.4016 (3)	0.0435 (16)	
C13	0.4773 (2)	0.7425 (6)	0.3584 (3)	0.0620 (19)	
H13A	0.4685	0.7557	0.3064	0.093*	
H13B	0.4490	0.7917	0.3731	0.093*	
H13C	0.4749	0.6476	0.3682	0.093*	
C14	0.4671 (2)	1.1580 (6)	0.3536 (4)	0.0498 (16)	
H14	0.4426	1.0829	0.3363	0.060*	
C15	0.4673 (3)	1.2560 (7)	0.3037 (4)	0.0596 (18)	
H15	0.4433	1.2476	0.2539	0.072*	
C16	0.5038 (3)	1.3675 (7)	0.3287 (4)	0.068 (2)	
H16	0.5058	1.4347	0.2960	0.082*	
C17	0.5371 (3)	1.3767 (7)	0.4030 (4)	0.068 (2)	
H17	0.5612	1.4517	0.4220	0.082*	
C18	0.5340 (3)	1.2731 (7)	0.4490 (4)	0.0594 (18)	
H18	0.5569	1.2796	0.4992	0.071*	
C19	0.6746 (3)	0.6130 (6)	0.3518 (4)	0.0611 (19)	
H19	0.6364	0.5870	0.3476	0.073*	
C20	0.7029 (3)	0.5394 (6)	0.3128 (4)	0.072 (2)	
H20	0.6833	0.4680	0.2823	0.087*	
C21	0.7591 (3)	0.5725 (8)	0.3196 (4)	0.074 (2)	
H21	0.7789	0.5247	0.2941	0.089*	
C22	0.7856 (3)	0.6764 (8)	0.3643 (4)	0.075 (2)	
H22	0.8245	0.7005	0.3707	0.091*	
C23	0.7548 (3)	0.7483 (7)	0.4012 (4)	0.069 (2)	
H23	0.7738	0.8203	0.4317	0.082*	
C24	0.1094 (2)	0.6327 (6)	0.5362 (3)	0.0374 (14)	
C25	0.2158 (2)	0.7108 (6)	0.5670 (3)	0.0423 (15)	
C26	0.1606 (2)	0.7229 (5)	0.5741 (3)	0.0379 (15)	
C27	0.1513 (2)	0.8231 (6)	0.6208 (3)	0.0480 (16)	
H27	0.1139	0.8306	0.6243	0.058*	
C28	0.1956 (3)	0.9092 (6)	0.6609 (3)	0.0580 (18)	
H28	0.1879	0.9766	0.6899	0.070*	
C29	0.2538 (3)	0.8954 (6)	0.6583 (3)	0.0482 (16)	
C30	0.2642 (2)	0.8003 (6)	0.6121 (3)	0.0435 (16)	
C31	0.3213 (3)	0.7890 (6)	0.6099 (3)	0.0526 (17)	
H31	0.3283	0.7261	0.5782	0.063*	
C32	0.3666 (3)	0.8676 (7)	0.6529 (4)	0.0618 (19)	
H32	0.4044	0.8576	0.6509	0.074*	
C33	0.3570 (3)	0.9630 (6)	0.6999 (4)	0.066 (2)	
H33	0.3883	1.0166	0.7298	0.079*	
C34	0.3015 (3)	0.9775 (6)	0.7020 (3)	0.0625 (19)	
H34	0.2949	1.0428	0.7328	0.075*	
C35	0.0757 (3)	0.3711 (6)	0.4118 (4)	0.0514 (17)	
C36	0.0298 (3)	0.2703 (6)	0.3697 (4)	0.076 (2)	
H36A	−0.0019	0.2692	0.3887	0.115*	
H36B	0.0476	0.1819	0.3752	0.115*	
H36C	0.0142	0.2948	0.3182	0.115*	
C37	−0.0701 (3)	0.5839 (7)	0.3374 (4)	0.0629 (19)	
H37	−0.0725	0.4904	0.3304	0.075*	
C38	−0.0951 (3)	0.6684 (9)	0.2760 (4)	0.078 (2)	
H38	−0.1141	0.6315	0.2291	0.093*	
C39	−0.0914 (3)	0.8031 (9)	0.2852 (5)	0.078 (2)	
H39	−0.1074	0.8605	0.2446	0.094*	
C40	−0.0644 (3)	0.8552 (8)	0.3536 (5)	0.075 (2)	
H40	−0.0621	0.9485	0.3610	0.090*	
C41	−0.0400 (3)	0.7670 (8)	0.4130 (4)	0.064 (2)	
H41	−0.0207	0.8036	0.4599	0.077*	
C42	0.2397 (3)	0.3316 (7)	0.3913 (4)	0.0597 (18)	
H42	0.2073	0.2743	0.3835	0.072*	
C43	0.2849 (3)	0.2897 (7)	0.3679 (4)	0.068 (2)	
H43	0.2824	0.2071	0.3438	0.082*	
C44	0.3327 (3)	0.3711 (8)	0.3810 (4)	0.071 (2)	
H44	0.3646	0.3426	0.3683	0.085*	
C45	0.3342 (3)	0.4934 (9)	0.4123 (4)	0.085 (2)	
H45	0.3659	0.5523	0.4193	0.102*	
C46	0.2876 (3)	0.5295 (7)	0.4337 (4)	0.068 (2)	
H46	0.2891	0.6138	0.4559	0.082*	

### Atomic displacement parameters (Å^2^)

**Table:**

	*U*^11^	*U*^22^	*U*^33^	*U*^12^	*U*^13^	*U*^23^
Cu1	0.0385 (6)	0.0540 (7)	0.0438 (7)	0.0005 (6)	0.0153 (5)	−0.0042 (6)
Cu2	0.0459 (5)	0.0645 (5)	0.0503 (5)	0.0058 (4)	0.0198 (4)	−0.0013 (5)
Cu3	0.0378 (6)	0.0606 (7)	0.0526 (7)	−0.0037 (6)	0.0178 (5)	−0.0091 (7)
Cu4	0.0426 (5)	0.0622 (5)	0.0497 (5)	−0.0004 (4)	0.0194 (4)	−0.0064 (5)
N1	0.024 (3)	0.053 (3)	0.042 (3)	0.004 (2)	0.012 (2)	0.005 (3)
N2	0.030 (3)	0.051 (3)	0.037 (3)	0.002 (2)	0.012 (2)	0.000 (3)
N3	0.039 (3)	0.055 (4)	0.042 (3)	−0.002 (3)	0.018 (3)	−0.005 (3)
N4	0.045 (3)	0.053 (4)	0.047 (4)	0.009 (3)	0.019 (3)	0.005 (3)
N5	0.033 (3)	0.047 (3)	0.036 (3)	−0.007 (2)	0.013 (2)	−0.006 (3)
N6	0.032 (3)	0.055 (3)	0.038 (3)	−0.007 (2)	0.012 (3)	−0.013 (3)
N7	0.038 (3)	0.063 (4)	0.052 (4)	0.001 (3)	0.020 (3)	−0.005 (3)
N8	0.044 (3)	0.051 (3)	0.046 (3)	0.001 (3)	0.022 (3)	0.003 (3)
O1	0.032 (2)	0.056 (3)	0.043 (3)	0.0000 (18)	0.016 (2)	−0.009 (2)
O2	0.034 (2)	0.068 (3)	0.044 (3)	−0.004 (2)	0.017 (2)	−0.005 (2)
O3	0.037 (2)	0.065 (3)	0.052 (3)	0.000 (2)	0.016 (2)	−0.015 (2)
O4	0.036 (2)	0.056 (3)	0.052 (3)	−0.007 (2)	0.022 (2)	−0.016 (2)
O5	0.035 (2)	0.058 (3)	0.051 (3)	−0.007 (2)	0.017 (2)	−0.015 (2)
O6	0.043 (3)	0.069 (3)	0.053 (3)	−0.009 (2)	0.023 (2)	−0.026 (2)
C1	0.035 (3)	0.039 (3)	0.034 (4)	0.006 (3)	0.007 (3)	0.013 (3)
C2	0.045 (4)	0.051 (4)	0.041 (4)	0.005 (4)	0.012 (3)	0.015 (4)
C3	0.037 (4)	0.045 (4)	0.036 (4)	0.000 (3)	0.010 (3)	0.004 (3)
C4	0.041 (4)	0.057 (4)	0.055 (5)	−0.011 (3)	0.017 (4)	−0.013 (4)
C5	0.064 (5)	0.070 (5)	0.058 (5)	−0.016 (4)	0.019 (4)	−0.017 (4)
C6	0.047 (4)	0.070 (5)	0.053 (5)	−0.009 (4)	0.015 (4)	0.001 (4)
C7	0.038 (4)	0.055 (4)	0.051 (4)	0.002 (3)	0.016 (4)	0.012 (4)
C8	0.049 (4)	0.083 (5)	0.067 (5)	−0.005 (4)	0.022 (4)	0.004 (4)
C9	0.032 (4)	0.114 (7)	0.088 (6)	−0.012 (4)	0.024 (4)	0.008 (5)
C10	0.042 (5)	0.113 (7)	0.083 (6)	−0.026 (5)	0.007 (5)	0.008 (6)
C11	0.050 (5)	0.092 (6)	0.066 (5)	−0.013 (4)	0.009 (4)	−0.008 (4)
C12	0.035 (4)	0.053 (4)	0.043 (4)	0.002 (3)	0.016 (3)	0.000 (4)
C13	0.043 (4)	0.079 (5)	0.066 (5)	−0.009 (3)	0.023 (4)	−0.028 (4)
C14	0.040 (4)	0.053 (4)	0.052 (5)	0.002 (3)	0.012 (4)	−0.003 (4)
C15	0.056 (5)	0.074 (5)	0.048 (5)	0.010 (4)	0.017 (4)	0.014 (4)
C16	0.074 (6)	0.069 (6)	0.070 (6)	0.019 (4)	0.037 (5)	0.035 (5)
C17	0.075 (5)	0.045 (4)	0.076 (6)	−0.008 (4)	0.018 (5)	0.007 (5)
C18	0.068 (5)	0.064 (5)	0.043 (4)	−0.005 (4)	0.017 (4)	−0.002 (4)
C19	0.061 (5)	0.054 (5)	0.075 (6)	0.008 (4)	0.034 (4)	−0.004 (4)
C20	0.080 (6)	0.072 (5)	0.071 (5)	0.014 (4)	0.035 (5)	−0.006 (4)
C21	0.079 (6)	0.083 (6)	0.076 (6)	0.032 (5)	0.048 (5)	0.009 (5)
C22	0.067 (5)	0.092 (6)	0.088 (6)	0.003 (5)	0.052 (5)	−0.018 (5)
C23	0.057 (5)	0.093 (6)	0.066 (5)	0.004 (4)	0.034 (4)	−0.012 (4)
C24	0.033 (4)	0.048 (4)	0.029 (4)	0.004 (3)	0.007 (3)	0.006 (3)
C25	0.037 (4)	0.043 (4)	0.044 (4)	0.006 (3)	0.012 (3)	0.007 (3)
C26	0.038 (4)	0.035 (4)	0.039 (4)	0.000 (3)	0.012 (3)	−0.006 (3)
C27	0.040 (4)	0.048 (4)	0.050 (4)	−0.001 (3)	0.008 (3)	−0.008 (4)
C28	0.065 (5)	0.051 (4)	0.058 (5)	0.004 (4)	0.022 (4)	−0.018 (4)
C29	0.043 (4)	0.053 (4)	0.043 (4)	−0.001 (3)	0.009 (3)	0.004 (4)
C30	0.039 (4)	0.047 (4)	0.042 (4)	−0.004 (3)	0.012 (3)	0.002 (3)
C31	0.045 (4)	0.052 (4)	0.061 (5)	−0.002 (3)	0.020 (4)	−0.007 (4)
C32	0.036 (4)	0.064 (5)	0.079 (5)	−0.004 (4)	0.012 (4)	−0.003 (4)
C33	0.049 (5)	0.058 (5)	0.073 (5)	−0.022 (4)	0.000 (4)	−0.010 (4)
C34	0.053 (5)	0.066 (5)	0.058 (5)	−0.010 (4)	0.009 (4)	−0.019 (4)
C35	0.039 (4)	0.063 (5)	0.049 (4)	−0.007 (3)	0.012 (4)	−0.015 (4)
C36	0.052 (5)	0.090 (6)	0.093 (6)	−0.014 (4)	0.034 (4)	−0.035 (5)
C37	0.064 (5)	0.071 (5)	0.058 (5)	0.003 (4)	0.029 (4)	−0.006 (5)
C38	0.067 (5)	0.118 (7)	0.047 (5)	0.012 (5)	0.019 (4)	−0.002 (6)
C39	0.062 (6)	0.093 (7)	0.082 (7)	0.018 (5)	0.029 (5)	0.024 (6)
C40	0.060 (5)	0.068 (6)	0.096 (7)	0.013 (4)	0.030 (5)	0.013 (6)
C41	0.049 (5)	0.070 (5)	0.071 (6)	0.003 (4)	0.021 (4)	−0.002 (5)
C42	0.058 (5)	0.056 (5)	0.070 (5)	0.006 (4)	0.030 (4)	−0.001 (4)
C43	0.087 (6)	0.060 (5)	0.073 (5)	0.029 (4)	0.048 (5)	0.013 (4)
C44	0.052 (5)	0.103 (6)	0.072 (6)	0.020 (4)	0.041 (4)	−0.001 (5)
C45	0.071 (5)	0.119 (7)	0.089 (6)	−0.022 (5)	0.058 (5)	−0.031 (6)
C46	0.069 (5)	0.076 (5)	0.072 (5)	−0.022 (4)	0.041 (4)	−0.025 (4)

### Geometric parameters (Å, °)

**Table:**

Cu1—O1	2.022 (3)	C13—H13B	0.9600
Cu1—O1^i^	2.022 (3)	C13—H13C	0.9600
Cu1—N2^i^	2.061 (4)	C14—C15	1.368 (7)
Cu1—N2	2.061 (4)	C14—H14	0.9300
Cu1—N3^i^	2.172 (5)	C15—C16	1.381 (8)
Cu1—N3	2.172 (5)	C15—H15	0.9300
Cu2—N1	1.832 (4)	C16—C17	1.371 (8)
Cu2—O2	1.838 (4)	C16—H16	0.9300
Cu2—O3	1.875 (4)	C17—C18	1.374 (8)
Cu2—N4	1.951 (5)	C17—H17	0.9300
Cu3—O4	2.025 (3)	C18—H18	0.9300
Cu3—O4^ii^	2.025 (3)	C19—C20	1.391 (7)
Cu3—N6^ii^	2.068 (4)	C19—H19	0.9300
Cu3—N6	2.068 (4)	C20—C21	1.351 (8)
Cu3—N7^ii^	2.176 (5)	C20—H20	0.9300
Cu3—N7	2.176 (5)	C21—C22	1.343 (8)
Cu4—O5	1.827 (4)	C21—H21	0.9300
Cu4—N5	1.828 (4)	C22—C23	1.395 (7)
Cu4—O6	1.861 (4)	C22—H22	0.9300
Cu4—N8	1.944 (4)	C23—H23	0.9300
N1—C1	1.330 (6)	C24—C26	1.479 (7)
N1—N2	1.420 (5)	C25—C26	1.390 (7)
N2—C12	1.292 (6)	C25—C30	1.469 (7)
N3—C14	1.324 (7)	C26—C27	1.412 (7)
N3—C18	1.327 (7)	C27—C28	1.363 (7)
N4—C19	1.307 (7)	C27—H27	0.9300
N4—C23	1.338 (6)	C28—C29	1.429 (7)
N5—C24	1.333 (6)	C28—H28	0.9300
N5—N6	1.412 (5)	C29—C30	1.380 (7)
N6—C35	1.307 (6)	C29—C34	1.407 (7)
N7—C41	1.327 (7)	C30—C31	1.396 (7)
N7—C37	1.339 (7)	C31—C32	1.349 (7)
N8—C46	1.324 (6)	C31—H31	0.9300
N8—C42	1.339 (7)	C32—C33	1.386 (8)
O1—C1	1.281 (5)	C32—H32	0.9300
O2—C2	1.312 (6)	C33—C34	1.360 (7)
O3—C12	1.318 (6)	C33—H33	0.9300
O4—C24	1.293 (5)	C34—H34	0.9300
O5—C25	1.316 (6)	C35—C36	1.488 (7)
O6—C35	1.310 (6)	C36—H36A	0.9600
C1—C3	1.483 (7)	C36—H36B	0.9600
C2—C3	1.390 (7)	C36—H36C	0.9600
C2—C7	1.484 (7)	C37—C38	1.396 (9)
C3—C4	1.401 (7)	C37—H37	0.9300
C4—C5	1.381 (7)	C38—C39	1.338 (9)
C4—H4	0.9300	C38—H38	0.9300
C5—C6	1.412 (7)	C39—C40	1.347 (9)
C5—H5	0.9300	C39—H39	0.9300
C6—C7	1.388 (8)	C40—C41	1.389 (8)
C6—C11	1.425 (8)	C40—H40	0.9300
C7—C8	1.386 (7)	C41—H41	0.9300
C8—C9	1.369 (8)	C42—C43	1.386 (7)
C8—H8	0.9300	C42—H42	0.9300
C9—C10	1.364 (9)	C43—C44	1.348 (8)
C9—H9	0.9300	C43—H43	0.9300
C10—C11	1.352 (8)	C44—C45	1.344 (8)
C10—H10	0.9300	C44—H44	0.9300
C11—H11	0.9300	C45—C46	1.377 (7)
C12—C13	1.507 (7)	C45—H45	0.9300
C13—H13A	0.9600	C46—H46	0.9300
			
O1—Cu1—O1^i^	180.0	C12—C13—H13A	109.5
O1—Cu1—N2^i^	100.79 (16)	C12—C13—H13B	109.5
O1^i^—Cu1—N2^i^	79.21 (16)	H13A—C13—H13B	109.5
O1—Cu1—N2	79.21 (16)	C12—C13—H13C	109.5
O1^i^—Cu1—N2	100.79 (16)	H13A—C13—H13C	109.5
N2^i^—Cu1—N2	180.0	H13B—C13—H13C	109.5
O1—Cu1—N3^i^	90.77 (16)	N3—C14—C15	123.6 (6)
O1^i^—Cu1—N3^i^	89.23 (16)	N3—C14—H14	118.2
N2^i^—Cu1—N3^i^	89.18 (16)	C15—C14—H14	118.2
N2—Cu1—N3^i^	90.82 (16)	C14—C15—C16	118.7 (6)
O1—Cu1—N3	89.23 (16)	C14—C15—H15	120.6
O1^i^—Cu1—N3	90.77 (16)	C16—C15—H15	120.6
N2^i^—Cu1—N3	90.82 (16)	C17—C16—C15	118.3 (6)
N2—Cu1—N3	89.18 (16)	C17—C16—H16	120.9
N3^i^—Cu1—N3	180.0	C15—C16—H16	120.9
N1—Cu2—O2	94.20 (19)	C16—C17—C18	118.9 (7)
N1—Cu2—O3	83.68 (18)	C16—C17—H17	120.6
O2—Cu2—O3	177.07 (17)	C18—C17—H17	120.6
N1—Cu2—N4	174.8 (2)	N3—C18—C17	123.2 (6)
O2—Cu2—N4	91.0 (2)	N3—C18—H18	118.4
O3—Cu2—N4	91.2 (2)	C17—C18—H18	118.4
O4—Cu3—O4^ii^	180.0	N4—C19—C20	123.4 (6)
O4—Cu3—N6^ii^	100.66 (16)	N4—C19—H19	118.3
O4^ii^—Cu3—N6^ii^	79.34 (16)	C20—C19—H19	118.3
O4—Cu3—N6	79.34 (16)	C21—C20—C19	119.5 (7)
O4^ii^—Cu3—N6	100.65 (16)	C21—C20—H20	120.2
N6^ii^—Cu3—N6	180.0	C19—C20—H20	120.2
O4—Cu3—N7^ii^	89.92 (18)	C22—C21—C20	118.0 (6)
O4^ii^—Cu3—N7^ii^	90.09 (18)	C22—C21—H21	121.0
N6^ii^—Cu3—N7^ii^	90.11 (17)	C20—C21—H21	121.0
N6—Cu3—N7^ii^	89.89 (17)	C21—C22—C23	120.2 (7)
O4—Cu3—N7	90.09 (18)	C21—C22—H22	119.9
O4^ii^—Cu3—N7	89.91 (18)	C23—C22—H22	119.9
N6^ii^—Cu3—N7	89.89 (17)	N4—C23—C22	122.0 (6)
N6—Cu3—N7	90.11 (17)	N4—C23—H23	119.0
N7^ii^—Cu3—N7	180.0	C22—C23—H23	119.0
O5—Cu4—N5	94.84 (18)	O4—C24—N5	122.4 (5)
O5—Cu4—O6	177.88 (16)	O4—C24—C26	119.6 (5)
N5—Cu4—O6	83.66 (18)	N5—C24—C26	118.0 (5)
O5—Cu4—N8	89.54 (19)	O5—C25—C26	125.3 (5)
N5—Cu4—N8	175.6 (2)	O5—C25—C30	116.7 (5)
O6—Cu4—N8	91.94 (19)	C26—C25—C30	118.1 (5)
C1—N1—N2	113.8 (4)	C25—C26—C27	120.2 (5)
C1—N1—Cu2	131.8 (4)	C25—C26—C24	123.6 (5)
N2—N1—Cu2	114.3 (4)	C27—C26—C24	116.2 (5)
C12—N2—N1	109.4 (4)	C28—C27—C26	121.9 (5)
C12—N2—Cu1	139.8 (4)	C28—C27—H27	119.1
N1—N2—Cu1	110.7 (3)	C26—C27—H27	119.1
C14—N3—C18	117.3 (6)	C27—C28—C29	119.7 (6)
C14—N3—Cu1	121.3 (5)	C27—C28—H28	120.2
C18—N3—Cu1	121.4 (4)	C29—C28—H28	120.2
C19—N4—C23	116.9 (5)	C30—C29—C34	118.7 (6)
C19—N4—Cu2	122.1 (4)	C30—C29—C28	119.9 (6)
C23—N4—Cu2	121.0 (5)	C34—C29—C28	121.4 (6)
C24—N5—N6	114.3 (4)	C29—C30—C31	119.1 (6)
C24—N5—Cu4	131.1 (4)	C29—C30—C25	120.1 (5)
N6—N5—Cu4	114.6 (3)	C31—C30—C25	120.7 (6)
C35—N6—N5	109.1 (4)	C32—C31—C30	121.3 (6)
C35—N6—Cu3	140.1 (4)	C32—C31—H31	119.4
N5—N6—Cu3	110.9 (3)	C30—C31—H31	119.4
C41—N7—C37	116.7 (6)	C31—C32—C33	120.3 (6)
C41—N7—Cu3	121.5 (5)	C31—C32—H32	119.8
C37—N7—Cu3	121.6 (5)	C33—C32—H32	119.8
C46—N8—C42	116.4 (5)	C34—C33—C32	119.4 (6)
C46—N8—Cu4	122.4 (4)	C34—C33—H33	120.3
C42—N8—Cu4	121.1 (4)	C32—C33—H33	120.3
C1—O1—Cu1	112.9 (3)	C33—C34—C29	121.1 (6)
C2—O2—Cu2	126.5 (3)	C33—C34—H34	119.4
C12—O3—Cu2	110.5 (4)	C29—C34—H34	119.4
C24—O4—Cu3	112.7 (3)	N6—C35—O6	121.4 (5)
C25—O5—Cu4	126.8 (3)	N6—C35—C36	120.6 (5)
C35—O6—Cu4	111.2 (4)	O6—C35—C36	118.0 (5)
O1—C1—N1	122.6 (5)	C35—C36—H36A	109.5
O1—C1—C3	119.4 (5)	C35—C36—H36B	109.5
N1—C1—C3	117.9 (5)	H36A—C36—H36B	109.5
O2—C2—C3	126.3 (5)	C35—C36—H36C	109.5
O2—C2—C7	116.3 (5)	H36A—C36—H36C	109.5
C3—C2—C7	117.4 (6)	H36B—C36—H36C	109.5
C2—C3—C4	120.5 (6)	N7—C37—C38	122.2 (7)
C2—C3—C1	122.9 (6)	N7—C37—H37	118.9
C4—C3—C1	116.5 (5)	C38—C37—H37	118.9
C5—C4—C3	121.9 (6)	C39—C38—C37	119.4 (8)
C5—C4—H4	119.1	C39—C38—H38	120.3
C3—C4—H4	119.1	C37—C38—H38	120.3
C4—C5—C6	119.9 (6)	C38—C39—C40	119.6 (8)
C4—C5—H5	120.1	C38—C39—H39	120.2
C6—C5—H5	120.1	C40—C39—H39	120.2
C7—C6—C5	119.8 (6)	C39—C40—C41	118.9 (8)
C7—C6—C11	119.3 (6)	C39—C40—H40	120.6
C5—C6—C11	120.8 (7)	C41—C40—H40	120.6
C8—C7—C6	118.7 (6)	N7—C41—C40	123.2 (7)
C8—C7—C2	121.1 (6)	N7—C41—H41	118.4
C6—C7—C2	120.2 (6)	C40—C41—H41	118.4
C9—C8—C7	121.1 (7)	N8—C42—C43	122.6 (6)
C9—C8—H8	119.5	N8—C42—H42	118.7
C7—C8—H8	119.5	C43—C42—H42	118.7
C10—C9—C8	120.3 (7)	C44—C43—C42	118.7 (7)
C10—C9—H9	119.9	C44—C43—H43	120.6
C8—C9—H9	119.9	C42—C43—H43	120.6
C11—C10—C9	121.0 (7)	C45—C44—C43	119.9 (6)
C11—C10—H10	119.5	C45—C44—H44	120.1
C9—C10—H10	119.5	C43—C44—H44	120.1
C10—C11—C6	119.6 (7)	C44—C45—C46	118.5 (7)
C10—C11—H11	120.2	C44—C45—H45	120.7
C6—C11—H11	120.2	C46—C45—H45	120.7
N2—C12—O3	122.0 (5)	N8—C46—C45	123.8 (6)
N2—C12—C13	120.4 (5)	N8—C46—H46	118.1
O3—C12—C13	117.6 (5)	C45—C46—H46	118.1
			
O2—Cu2—N1—C1	−7.1 (5)	N1—C1—C3—C4	−177.5 (5)
O3—Cu2—N1—C1	175.0 (5)	C2—C3—C4—C5	2.9 (9)
N4—Cu2—N1—C1	164.5 (19)	C1—C3—C4—C5	−179.0 (5)
O2—Cu2—N1—N2	175.8 (3)	C3—C4—C5—C6	1.1 (9)
O3—Cu2—N1—N2	−2.2 (3)	C4—C5—C6—C7	−1.5 (9)
N4—Cu2—N1—N2	−13 (2)	C4—C5—C6—C11	174.9 (6)
C1—N1—N2—C12	−176.7 (5)	C5—C6—C7—C8	178.0 (6)
Cu2—N1—N2—C12	1.0 (5)	C11—C6—C7—C8	1.6 (9)
C1—N1—N2—Cu1	5.7 (5)	C5—C6—C7—C2	−1.9 (9)
Cu2—N1—N2—Cu1	−176.6 (2)	C11—C6—C7—C2	−178.3 (5)
O1—Cu1—N2—C12	176.3 (6)	O2—C2—C7—C8	4.1 (8)
O1^i^—Cu1—N2—C12	−3.7 (6)	C3—C2—C7—C8	−174.2 (5)
N3^i^—Cu1—N2—C12	85.7 (6)	O2—C2—C7—C6	−176.0 (5)
N3—Cu1—N2—C12	−94.3 (6)	C3—C2—C7—C6	5.6 (8)
O1—Cu1—N2—N1	−7.2 (3)	C6—C7—C8—C9	−3.1 (9)
O1^i^—Cu1—N2—N1	172.8 (3)	C2—C7—C8—C9	176.7 (6)
N3^i^—Cu1—N2—N1	−97.8 (3)	C7—C8—C9—C10	2.9 (11)
N3—Cu1—N2—N1	82.2 (3)	C8—C9—C10—C11	−0.9 (12)
O1—Cu1—N3—C14	151.1 (4)	C9—C10—C11—C6	−0.6 (11)
O1^i^—Cu1—N3—C14	−28.9 (4)	C7—C6—C11—C10	0.3 (10)
N2^i^—Cu1—N3—C14	−108.1 (4)	C5—C6—C11—C10	−176.1 (6)
N2—Cu1—N3—C14	71.9 (4)	N1—N2—C12—O3	1.7 (7)
O1—Cu1—N3—C18	−26.9 (4)	Cu1—N2—C12—O3	178.2 (4)
O1^i^—Cu1—N3—C18	153.1 (4)	N1—N2—C12—C13	−179.3 (5)
N2^i^—Cu1—N3—C18	73.9 (4)	Cu1—N2—C12—C13	−2.8 (9)
N2—Cu1—N3—C18	−106.1 (4)	Cu2—O3—C12—N2	−3.4 (6)
N1—Cu2—N4—C19	−1(2)	Cu2—O3—C12—C13	177.6 (4)
O2—Cu2—N4—C19	170.2 (5)	C18—N3—C14—C15	0.9 (9)
O3—Cu2—N4—C19	−11.8 (5)	Cu1—N3—C14—C15	−177.2 (4)
N1—Cu2—N4—C23	177 (2)	N3—C14—C15—C16	0.4 (9)
O2—Cu2—N4—C23	−11.3 (5)	C14—C15—C16—C17	−1.8 (9)
O3—Cu2—N4—C23	166.7 (5)	C15—C16—C17—C18	1.8 (10)
O5—Cu4—N5—C24	5.5 (5)	C14—N3—C18—C17	−0.8 (9)
O6—Cu4—N5—C24	−176.0 (5)	Cu1—N3—C18—C17	177.3 (4)
N8—Cu4—N5—C24	178 (60)	C16—C17—C18—N3	−0.6 (10)
O5—Cu4—N5—N6	−176.4 (3)	C23—N4—C19—C20	−2.5 (9)
O6—Cu4—N5—N6	2.1 (3)	Cu2—N4—C19—C20	176.0 (5)
N8—Cu4—N5—N6	−4(3)	N4—C19—C20—C21	1.9 (10)
C24—N5—N6—C35	176.4 (5)	C19—C20—C21—C22	0.0 (11)
Cu4—N5—N6—C35	−2.0 (5)	C20—C21—C22—C23	−1.0 (11)
C24—N5—N6—Cu3	−4.1 (5)	C19—N4—C23—C22	1.3 (9)
Cu4—N5—N6—Cu3	177.5 (2)	Cu2—N4—C23—C22	−177.2 (5)
O4—Cu3—N6—C35	−175.5 (7)	C21—C22—C23—N4	0.4 (11)
O4^ii^—Cu3—N6—C35	4.5 (7)	Cu3—O4—C24—N5	5.6 (6)
N7^ii^—Cu3—N6—C35	94.6 (6)	Cu3—O4—C24—C26	−173.7 (4)
N7—Cu3—N6—C35	−85.4 (6)	N6—N5—C24—O4	−0.9 (7)
O4—Cu3—N6—N5	5.2 (3)	Cu4—N5—C24—O4	177.2 (4)
O4^ii^—Cu3—N6—N5	−174.8 (3)	N6—N5—C24—C26	178.4 (4)
N7^ii^—Cu3—N6—N5	−84.7 (3)	Cu4—N5—C24—C26	−3.5 (7)
N7—Cu3—N6—N5	95.3 (3)	Cu4—O5—C25—C26	−2.1 (8)
O4—Cu3—N7—C41	−26.7 (4)	Cu4—O5—C25—C30	178.9 (3)
O4^ii^—Cu3—N7—C41	153.3 (4)	O5—C25—C26—C27	−176.0 (5)
N6^ii^—Cu3—N7—C41	73.9 (4)	C30—C25—C26—C27	2.9 (8)
N6—Cu3—N7—C41	−106.1 (4)	O5—C25—C26—C24	5.6 (9)
N7^ii^—Cu3—N7—C41	153 (100)	C30—C25—C26—C24	−175.4 (5)
O4—Cu3—N7—C37	148.8 (4)	O4—C24—C26—C25	176.6 (5)
O4^ii^—Cu3—N7—C37	−31.2 (4)	N5—C24—C26—C25	−2.7 (8)
N6^ii^—Cu3—N7—C37	−110.6 (5)	O4—C24—C26—C27	−1.8 (7)
N6—Cu3—N7—C37	69.4 (5)	N5—C24—C26—C27	178.9 (5)
N7^ii^—Cu3—N7—C37	−31 (100)	C25—C26—C27—C28	−0.9 (9)
O5—Cu4—N8—C46	−17.8 (5)	C24—C26—C27—C28	177.5 (5)
N5—Cu4—N8—C46	170 (2)	C26—C27—C28—C29	−2.4 (9)
O6—Cu4—N8—C46	163.7 (5)	C27—C28—C29—C30	3.7 (9)
O5—Cu4—N8—C42	159.7 (5)	C27—C28—C29—C34	−176.5 (6)
N5—Cu4—N8—C42	−13 (3)	C34—C29—C30—C31	−0.2 (8)
O6—Cu4—N8—C42	−18.8 (5)	C28—C29—C30—C31	179.6 (5)
N2^i^—Cu1—O1—C1	−172.1 (3)	C34—C29—C30—C25	178.6 (5)
N2—Cu1—O1—C1	7.9 (3)	C28—C29—C30—C25	−1.6 (8)
N3^i^—Cu1—O1—C1	98.6 (4)	O5—C25—C30—C29	177.4 (5)
N3—Cu1—O1—C1	−81.4 (4)	C26—C25—C30—C29	−1.6 (8)
N1—Cu2—O2—C2	5.0 (5)	O5—C25—C30—C31	−3.8 (8)
O3—Cu2—O2—C2	48 (3)	C26—C25—C30—C31	177.2 (5)
N4—Cu2—O2—C2	−174.3 (4)	C29—C30—C31—C32	1.0 (9)
N1—Cu2—O3—C12	2.9 (4)	C25—C30—C31—C32	−177.8 (6)
O2—Cu2—O3—C12	−41 (3)	C30—C31—C32—C33	−0.7 (10)
N4—Cu2—O3—C12	−178.0 (4)	C31—C32—C33—C34	−0.5 (10)
N6^ii^—Cu3—O4—C24	174.3 (3)	C32—C33—C34—C29	1.3 (10)
N6—Cu3—O4—C24	−5.7 (3)	C30—C29—C34—C33	−0.9 (9)
N7^ii^—Cu3—O4—C24	84.2 (4)	C28—C29—C34—C33	179.3 (6)
N7—Cu3—O4—C24	−95.8 (4)	N5—N6—C35—O6	0.6 (8)
N5—Cu4—O5—C25	−2.6 (4)	Cu3—N6—C35—O6	−178.7 (4)
O6—Cu4—O5—C25	−48 (5)	N5—N6—C35—C36	179.9 (5)
N8—Cu4—O5—C25	178.0 (4)	Cu3—N6—C35—C36	0.6 (10)
O5—Cu4—O6—C35	44 (5)	Cu4—O6—C35—N6	1.0 (7)
N5—Cu4—O6—C35	−1.7 (4)	Cu4—O6—C35—C36	−178.3 (5)
N8—Cu4—O6—C35	177.8 (4)	C41—N7—C37—C38	−0.8 (9)
Cu1—O1—C1—N1	−7.6 (6)	Cu3—N7—C37—C38	−176.5 (4)
Cu1—O1—C1—C3	171.2 (4)	N7—C37—C38—C39	0.7 (10)
N2—N1—C1—O1	1.2 (7)	C37—C38—C39—C40	−0.9 (10)
Cu2—N1—C1—O1	−176.0 (4)	C38—C39—C40—C41	1.2 (11)
N2—N1—C1—C3	−177.6 (4)	C37—N7—C41—C40	1.2 (9)
Cu2—N1—C1—C3	5.2 (8)	Cu3—N7—C41—C40	176.9 (5)
Cu2—O2—C2—C3	−1.6 (8)	C39—C40—C41—N7	−1.4 (10)
Cu2—O2—C2—C7	−179.9 (3)	C46—N8—C42—C43	0.6 (9)
O2—C2—C3—C4	175.7 (5)	Cu4—N8—C42—C43	−177.0 (5)
C7—C2—C3—C4	−6.0 (8)	N8—C42—C43—C44	1.7 (10)
O2—C2—C3—C1	−2.2 (9)	C42—C43—C44—C45	−3.7 (11)
C7—C2—C3—C1	176.0 (5)	C43—C44—C45—C46	3.2 (11)
O1—C1—C3—C2	−178.3 (5)	C42—N8—C46—C45	−1.1 (10)
N1—C1—C3—C2	0.5 (8)	Cu4—N8—C46—C45	176.5 (5)
O1—C1—C3—C4	3.7 (7)	C44—C45—C46—N8	−0.8 (11)

Symmetry codes: (i) −*x*+1, −*y*+2, −*z*+1; (ii) −*x*, −*y*+1, −*z*+1.

## References

[bb1] Bruker (2003). *SMART*, *SAINT* and *SADABS* Bruker AXS Inc., Madison, Wisconsin, USA.

[bb2] Patole, J., Sandbhor, U., Padhye, S., Deobagkar, D. N., Anson, C. E. & Powell, A. (2003). *Bioorg. Med. Chem. Lett.* **13**, 51–55. 10.1016/s0960-894x(02)00855-7 12467615

[bb3] Pouralimardan, O., Chamayou, A. C., Janiak, C. & Hassan, H. M. (2007). *Inorg. Chim. Acta*, **360**, 1599–1608.

[bb4] Sheldrick, G. M. (2008). *Acta Cryst.* A**64**, 112–122. 10.1107/S0108767307043930 18156677

